# Computational pathology definitions, best practices, and recommendations for regulatory guidance: a white paper from the Digital Pathology Association

**DOI:** 10.1002/path.5331

**Published:** 2019-09-03

**Authors:** Esther Abels, Liron Pantanowitz, Famke Aeffner, Mark D Zarella, Jeroen van der Laak, Marilyn M Bui, Venkata NP Vemuri, Anil V Parwani, Jeff Gibbs, Emmanuel Agosto‐Arroyo, Andrew H Beck, Cleopatra Kozlowski

**Affiliations:** ^1^ Regulatory and Clinical Affairs PathAI Boston MA USA; ^2^ Department of Pathology University of Pittsburgh Medical Center Pittsburgh PA USA; ^3^ Amgen Research, Comparative Biology and Safety Sciences Amgen Inc. South San Francisco CA USA; ^4^ Department of Pathology and Laboratory Medicine Drexel University College of Medicine Philadelphia PA USA; ^5^ Department of Pathology Radboud University Medical Center Nijmegen The Netherlands; ^6^ Center for Medical Image Science and Visualization Linköping University Linköping Sweden; ^7^ Department of Pathology Moffitt Cancer Center Tampa FL USA; ^8^ Data Science Department Chan Zuckerberg Biohub San Francisco CA USA; ^9^ Department of Pathology The Ohio State University Columbus OH USA; ^10^ Hyman, Phelps & McNamara, P.C Washington DC USA; ^11^ PathAI Boston MA USA; ^12^ Department of Development Sciences Genentech Inc. South San Francisco CA USA

**Keywords:** artificial intelligence, computational pathology, convolutional neural networks, digital pathology, deep learning, image analysis, machine learning

## Abstract

In this white paper, experts from the Digital Pathology Association (DPA) define terminology and concepts in the emerging field of computational pathology, with a focus on its application to histology images analyzed together with their associated patient data to extract information. This review offers a historical perspective and describes the potential clinical benefits from research and applications in this field, as well as significant obstacles to adoption. Best practices for implementing computational pathology workflows are presented. These include infrastructure considerations, acquisition of training data, quality assessments, as well as regulatory, ethical, and cyber‐security concerns. Recommendations are provided for regulators, vendors, and computational pathology practitioners in order to facilitate progress in the field. © 2019 The Authors. *The Journal of Pathology* published by John Wiley & Sons Ltd on behalf of Pathological Society of Great Britain and Ireland.

## Introduction: goals of this paper

The term **computational pathology (CPATH)** has become a buzz‐word among the **digital pathology** community, yet it often leads to confusion due to its use in different contexts 1, 2, 3. The expert authors of the Digital Pathology Association (DPA) define CPATH as the ‘omics’ or ‘big‐data’ approach to pathology, where multiple sources of patient information including pathology image data and **meta‐data** are combined to extract patterns and analyze features. In this white paper, we will focus on a subset of this field, encompassing CPATH applications related to **whole slide imaging (WSI)** and analysis. CPATH is only one of a large number of fashionable terms that are confusingly used apparently interchangeably, yet mean somewhat different things. To assist the reader, we have employed specialized terminology in bold case when they first appear, and provided a short definition of each in Table 1.

**Table 1:** Definitions of CPATH terms

**Annotation**	Indication of the position and/or outline of structures or objects within digital images, usually produced by humans using a computer mouse or drawing tablet. Annotations may have associated labels and possible other meta‐data. Annotations can be manually generated or can be established by algorithm tools
**Artificial intelligence (AI)**	A branch of computer science dealing with the simulation of intelligent behavior in computers
**Black box/glass box**	A neural network can be perceived as a black box that lacks a clear depiction of the image features used for a decision. However, methods can be employed to transform it into a glass box in an effort to understand the relationship between the input parameters and the output of the network
**Cloud computing**	The practice of using a network of remote servers hosted on the internet to store, manage, and process data, rather than a local server or a personal computer
**Computational pathology (CPATH)**	A branch of pathology that involves computational analysis of a broad array of methods to analyze patient specimens for the study of disease. In this paper, we focus on the extraction of information from digitized pathology images in combination with their associated meta‐data, typically using AI methods such as deep learning
**Convolutional neural network (CNN)**	A type of deep neural network particularly designed for images. It uses a kernel or filter to convolve an image, which results in features useful for differentiating images
**Data augmentation**	Method commonly used in deep learning to increase the training data using operations such as rotating, cropping, zooming, and image histogram‐based modifications. This provides a number of advantages such as promoting positional and rotational invariance, robustness to staining variability, and improves the generalizability of the classifier
**Deep learning**	The subset of machine learning composed of algorithms that permit software to train itself to perform tasks by exposing multilayered artificial neural networks to vast amounts of data. Data are fed into the input layers and are sequentially processed in a hierarchical manner with increasing complexity at each layer, modeled loosely after the hierarchical organization in the brain. Optimization functions are iteratively trained to shape the processing functions of the layers and the connections between them
**Digital pathology**	A blanket term that encompasses tools and systems to digitize pathology slides and associated meta‐data, their storage, review, analysis, and enabling infrastructure
**Gold standard**	The practical standard that is used to capture the ‘ground truth’. The gold standard may not always be perfectly correct, but in general is viewed as the best approximation
**Ground truth** (as considered within AI)	A category, quantity, or label assigned to a dataset that provides guidance to an algorithm during training. Depending on the task, the ground truth can be a patient‐ or slide‐level characterization or can be applied to objects or regions within the image. The ground truth is an abstract concept of the ‘truth’
**Image analysis**	A method to extract typically quantifiable information from images. In this paper, we only discuss image analysis as applied to images of histology slides, but the term itself is broader, and applies to the extraction of information from any image, biomedical or not
**Machine learning (ML)**	A branch of AI in which computer software learns to perform a task by being exposed to representative data
**Meta‐data**	In the context of digital pathology, the term meta‐data describes descriptive data associated with the individual, sample, or slide. They may include image acquisition information, patient demographic data, pathologist annotation or classification, or outcome data from treatment. Typically, meta‐data are entries that allow searches in databases, for example. Highly complex, large, multiple‐time‐point associated data, such as longitudinal image data (such as radiology) or genomic data, are not usually called ‘meta‐data’
**Supervised machine learning**	Supervised learning is used to train a model to predict an outcome or to classify a dataset based on a label associated with a data point (i.e. ground truth). An example of supervised machine learning includes the design of classifiers to distinguish benign from malignant regions based on manual annotations
**Unsupervised machine learning**	Unsupervised learning seeks to identify natural divisions in a dataset without the need for a ground truth, often using methods such as cluster analysis or pattern matching. Examples of unsupervised machine learning include the identification of images with similar attributes or the clustering of tumors into subtypes
**Whole slide image**	Digital representation of an entire histopathological glass slide, digitized at microscope resolution. These whole slide scans are typically produced using slide scanners. Slide scan viewing software enables inspection of the image in a way that mimics the use of a traditional microscope; the image can be viewed at different magnifications

As this review will discuss, the use of advanced computational techniques such as **machine learning (ML)**, and in particular **deep learning (DL)**, has been a key element in the promise of CPATH. Both ML and its subset DL are examples of **artificial intelligence (AI)**. A related concept is ML‐powered **image analysis**, which allows extremely accurate image classification or segmentation of an image. The outputs of these computer‐based tools may later be integrated into a full CPATH process, once these image features are correlated to other types of patient information besides the image itself. Whilst this type of analysis holds great promise for a paradigm‐shift in healthcare, multiple barriers remain, precluding its widespread clinical use.

The goal of this white paper is to facilitate the understanding of CPATH by the pathology community and regulators to enable a stronger collaboration with the CPATH industry. We discuss the history of CPATH and best practices in deploying these methods for clinical use, as well as technical and regulatory considerations, design controls, and concerns. Finally, recommendations and future directions to help drive the field forward are discussed.

## History and promises of computational pathology

### Introduction of whole slide imaging

Histopathology has been an integral part of the work of pathologists since the 17th century 4. Today, histopathology largely remains a manual process in which pathologists examine glass slides using conventional brightfield microscopy. Advances in digitization of glass slides in pathology occurred much later than the digital transformation witnessed in radiology 5, 6, where digital sensors are widespread. When histologic glass slides are digitized, they can be remotely viewed by a pathologist on a computer screen, or digitally analyzed using **image analysis** techniques 7.

At its inception, digital image analysis was predominantly used by researchers and often limited to individual fields of views, which was cumbersome and could introduce bias. WSI allowed developments that have brought us from the application of traditional image analysis techniques on small manually selected regions of interest, to what is the current state‐of‐the‐art in digital pathology: techniques that process the entire slide image automatically 7. This allowed researchers to identify features not easily analyzed by visual evaluation alone 8, 9.

Various whole slide scanners with brightfield and fluorescent capabilities have entered the market 10. In the European Union, several WSI devices are marketed for clinical use, whilst in the United States, only two WSI devices have been cleared by regulators for primary diagnosis 11, 12. Whole slide image analysis techniques are now routinely utilized for basic and translational research, drug development, and clinical diagnostics including laboratory‐developed tests and *in vitro* diagnostics.

### Traditional image analysis enhanced by machine learning

Traditional digital image analysis focuses on three broad categories of measurements: localization, classification, and quantification of image objects. This method is an iterative process where typically a few parameters are manually tuned, built into an algorithm, and often tested only on a region of the slide image 13. Aspects that fail a quality control review are tweaked until the algorithm performance meets pre‐determined analysis criteria.

ML has facilitated significant advancements within the field of image analysis, as it often allows the generation of more robust algorithms that need fewer iterative optimizations for each dataset, compared with methods where parameters are manually tuned. **Supervised** ML techniques, in which an algorithm is trained using **ground truth labels**, are particularly effective in image segmentation (detection of specific objects) and classification (such as tumor diagnosis) tasks 14. The ground truths may be a category or label assigned to a dataset that provides guidance to an algorithm.

The capabilities of ML have dramatically expanded in the last decade due to the developments in **deep learning** 15, an approach that enables an algorithm to automatically discover relevant image features that contribute to computer‐vision tasks. One of the first uses of deep learning in histopathology was the work of Ciresan *et al* in the International Conference on Pattern Recognition (ICPR) challenge in 2012 16, which focused on fully automated recognition of mitotic figures in hematoxylin and eosin (H&E)‐stained breast cancer tissue 17. Using **convolutional neural networks (CNNs)**, a type of deep learning algorithm, the authors were able to generate results that far exceeded those of the competition.

These early studies applied ML to histopathology using small, manually selected regions of interest, but later research showed that these techniques could work equally well on whole slide images 18. In DP, ML‐enhanced image analysis is now widely employed by researchers and implemented in a number of commercially available image analysis software products.

### Correlating images to patient outcome

Researchers quickly found that ML algorithms could be used in novel ways that were not limited to information contained only in the slide image. ML algorithms could be used to extract an enormous number of features in an image. For example, ML‐powered image analysis can be used to identify objects in a histology image which may be used to generate ‘histologic primitives’ such as nuclei, tumor cells, etc. that a human would consider an ‘object’ 14. When these features are correlated to non‐image patient features from the medical record, such as response to specific treatment, algorithms can be developed that may predict these responses from images alone. The image features need not necessarily be ‘objects’ that may be recognized by a human. For example, Beck *et al* examined ‘a rich quantitative feature set’ consisting of 6642 engineered features expressing characteristics of both breast cancer epithelium and stroma 19 and correlated these to patient outcome. They identified a small number of stromal morphological features that yielded mutually independent prognostic information about breast cancer. It is possible to go a step further, and train a deep learning algorithm in such a way that an enormous number of image features are automatically extracted and used to obtain patient outcome‐related predictions from images 20. There is a trade‐off: the larger the number and level of abstraction of image features used for the predictions, the greater the difficulty in understanding those predictions (see our section on understanding algorithms).

Nevertheless, these types of analyses are what many consider to be the biggest promise of CPATH, particularly in the field of oncology, where it may increase the speed and accuracy of diagnosis.

### Assisting diagnosis

One of the promises of CPATH is that it can be used to build a clinical decision support tool for precision diagnosis of the patient. For example, algorithms have been described that identify images likely to contain tumor cells 21, compute mitotic counts 22, improve the accuracy and precision of immunohistochemistry scoring 23, 24, or apply standardized histological scoring criteria such as the Gleason score 25, which can be critically important in the management of cancer and guiding treatment strategy 26. Spatial relationships among immune cells within the stromal or tumor compartments of the tumor microenvironment can also be evaluated using deep learning tools and correlated to response with immunotherapy 27.

Another widely studied application is detection of lymph node metastases 28, for which it was shown that the use of a deep learning algorithm improved sensitivity with equal specificity, while requiring significantly less time for diagnostics 29. An ideal decision support tool would incorporate such algorithms into a user‐friendly system, aiding clinicians in making the best treatment decisions, while avoiding information overload and decision paralysis 30, 31.

### Identifying novel features

The capacity of ML to identify new image features may lead to the discovery of previously unrecognized morphological characteristics with clinical relevance that have not been used in visual assessment by pathologists, either because these features had not previously been discovered or because they are beyond human visual perception. For example, deep learning is capable of assessing morphological information from the stroma neighboring ductal carcinoma *in situ* (DCIS) breast lesions which correlates with DCIS grade 32. It should be noted that even though pathologists recognize morphological changes as a consequence of the presence of tumor, they may not currently directly use this information in diagnostics or to offer prognostic insight. The fact that deep learning is capable of using such ‘hidden’ features is promising as it may yield prognostic information not currently utilized. Once identified, it may be possible to re‐engineer a simpler image analysis algorithm to identify the specific feature, which may be more easily accepted by clinicians compared to the deep learning algorithm.

## Hurdles and solutions for implementing computational pathology

Despite the promises of CPATH, most algorithms used in current clinical practice are limited to traditional image analysis of immunohistochemical stains, which do not employ advanced ML techniques such as deep learning. In this section, we address the many barriers to implementing CPATH for clinical use, and potential strategies to overcome them.

### Infrastructure considerations

Implementation of CPATH may require a significant investment in IT infrastructure. In general, data to be analyzed are captured as images of tissue sections, often scanned at 20× or 40× objective magnification. In clinical practice, pathology images are commonly larger than 50 000 by 50 000 pixels 33. As a benchmark, this can translate into estimated file sizes ranging from 0.5 to 4 GB for 40× images, depending on the size of the scan area and image compression type.

The large size of these images may present a problem for evaluation, storage, and inventory management. The primary computing obstacles that users face are processor speed and memory requirements of local workstations, data storage requirements, and limitations of the network. For CPATH to perform effectively, it is important that there are safeguards to ensure that images are fully loaded and that the analysis algorithm is not interrupted due to insufficient bandwidth, processing power or memory. Additional considerations when running deep learning algorithms include, but are not limited to, the number of intended users, flexibility of the server or cloud configuration to accommodate new algorithms or case‐loads, cyber‐security, and associated costs.

### Processor speed and sources

The performance of any image processing is highly dependent on processor speed 34. Deep learning is best performed using graphics processing units (GPUs), which can provide significant performance enhancement over central processing units (CPUs) 35. Most computers are designed to perform computations on their CPU and use the GPU simply to render graphics. It may be necessary to purchase a more powerful GPU designed for deep learning; these are generally more expensive and tend to generate more heat. Some laboratories may therefore elect to dedicate high‐performance workstations strictly for deep learning. However, some vendors offer the ability to perform image analysis at the server or cloud level, which may provide significantly more resources and can potentially distribute deep learning capabilities to a much larger user base.

### Network limitations

For implementations in which either data are stored remotely or image processing is performed remotely, network bandwidth becomes an important consideration. The large size of whole slide images presents a potential hurdle for efficient processing in environments that lack sufficient bandwidth. Depending on the network implementation, there are several data transfer considerations. First, digital slide data from the whole slide scanner must be transferred to its network storage location, which requires the file in its entirety. Second, the digital slide must be transferred from its network storage location to the image analysis environment (which may reside locally, elsewhere on the network, or in the cloud), which can often be accomplished in a more efficient manner, since the entire image is unlikely to be analyzed at once. Training a deep learning network on an entire slide image at full resolution is currently very challenging, so it usually operates on a smaller tiled image or patch 33. Downscaling (reducing resolution) of these images is one possible approach, but this may lead to loss of discriminative details as using small, high‐resolution tiles may lose tissue context. The optimal resolution and tile size for analysis are highly case‐dependent 36. If only small regions of interest are to be processed, or if the processing can occur at a reduced magnification, smaller portions of the virtual slide file need to be transferred due to the pyramid structure of most WSI file formats 37.

### Acquiring training data

Deep learning is generally extremely data‐hungry, especially compared with traditional image analysis where ‘important features’ are manually selected, as it must automatically identify these features. For supervised learning, in addition to the raw image sets, a **ground truth** must be included in the dataset to provide appropriate diagnostic context. Algorithms can then be trained to predict or characterize an image guided by the ground truth provided. The ground truth may be derived from patient outcome data, a field extracted from the pathology report or laboratory information system (e.g. histologic grade), a quantitative score assigned to the case (e.g. molecular testing), or it can be a factor manually provided by a pathologist reviewing the case specifically to support the algorithm training. Obtaining clinical ground truth data suitable for algorithm development is often time‐consuming and challenging. It usually takes a long time to generate enough survival data from clinical patients, and clinical data are generally locked into an unstructured format within one or more disparate electronic medical records. The data need to be manually or automatically 38 curated before being incorporated into an algorithm.

Furthermore, training may also require manual annotations applied to the digital slide, including the designation of specific areas of interest, for instance identifying cancer from benign tissue.

Obtaining adequately annotated datasets for deep learning by a trained expert can be difficult 39 due to the amount of time required, associated expenses, and the tedious nature of the task. The use of streamlined workflows and a single common annotation tool with an intuitive user interface can make the task of creating and sharing manual regional annotations considerably easier. Web‐based tools may be ideal in sharing annotations between different research groups, as they avoid the need to install specific software on multiple systems 40. In addition, research has shown that, for some tasks, annotations of expert observers (i.e. pathologists) may not always be necessary 41 (see the section on crowdsourcing). Yet there is generally a trade‐off between quantity and accuracy. Also, training images must be representative of the images that the algorithm is designed to be applied to, and appropriately ‘balanced’; for example, contain approximately similar numbers of examples for different objects it is intended to identify.

### Data variability

It is important that supervised algorithms are developed using a wide variety of data sources, to more robustly handle variations when exposed to other datasets. A consideration is to implement prospective review using retrospective data during development, and/or verification or validation. When algorithms are developed using limited datasets supplied by only one or few pathology laboratories, the algorithms may not have incorporated all of the variations and artifacts encountered across different labs, including pre‐imaging, imaging, and post‐imaging steps within the WSI workflow. This is in part because, in surgical pathology, there is currently no accepted global standard for tissue processing, staining, and slide preparation. Even digital acquisition may introduce variability 42. As such, an algorithm designed to perform well on one set of WSIs may not perform equally well when generalized and used around the world by many laboratories. This could be somewhat alleviated by implementing consistent pre‐imaging steps, applying manual or automated image quality control processes, using larger and more representative training sets, and calibrating algorithms for each lab prior to being used for clinical work. It is also possible to apply image pre‐processing strategies such as color normalization 43 to reduce the impact of stain and processing variability, and **data augmentation** to artificially add variation and increase (or balance) the training data to make them more representative of the application data. Other best practices include testing developed models using a variety of test and validation sets to avoid over‐fitting, and clearly reporting characteristics of the patients used to build a model, since additional training data may be required for it to perform well on other populations. One may consider the addition of prospective real‐world data collection to monitor and optimize performance.

### Public sources

There are currently only limited publicly available datasets with annotated images and associated non‐image patient data that are required for CPATH. This may be one of the greatest factors limiting progress in the field of CPATH. However, some initiatives to overcome these hurdles are described below. The Cancer Genome Atlas (TCGA) has performed comprehensive molecular profiling on approximately 10 000 cancers (National Cancer Institute, The Cancer Genome Atlas, https://cancergenome.nih.gov/). In addition to the collection of molecular and clinical data, TCGA has collected WSI data from a subset of its participants 39. Some other examples of public digital slide datasets are the breast cancer images used for the CAMELYON competition 44; the Medical Image Computing and Computer Assisted Intervention Society (MICCAI) 2014 brain tumor digital pathology challenge for distinguishing brain cancer subtypes 45; and the Tumor Proliferation Assessment Challenge (TUPAC16), which includes hundreds of cases 46. The Grand Challenge website (https://grand-challenge.org/challenges/) maintains a list of all challenges that have been organized in the field of medical image analysis. Some of these challenges offer developers additional pathology digital datasets for CPATH. However, care must be taken as the quality of public samples may be variable, so they should be carefully tested before use.

### Crowdsourcing

An alternative for obtaining large‐scale image annotations is crowdsourcing, in which this function is outsourced to an undefined and generally large group of non‐expert people in the form of an open call 47. Crowdsourced image annotation has been successfully used to serve a diverse set of scientific goals 40, including detection of malaria from blood smears 39, and estrogen receptor classification 47. Compared with public sources or pathologist annotations, crowdsourcing may be cheaper and quicker, but has the potential to introduce noise 33. It is possible that this noise can be compensated by a sufficiently large body of training data 48, and by having multiple people annotate the same slide to achieve consensus. But it is imperative to ensure that all annotators are taught to perform the task in the same way.

### Active learning

Active learning is considered semi‐supervised learning, which may reduce the size of required training data 49. In active learning, the algorithm interactively queries for expert assistance to obtain annotations for ambiguous data points. Essentially, the algorithm uses a sampling strategy to select small sets of data iteratively for experts to label, only when it has trouble determining the outcome. For each iteration, the classifier is updated and then all unlabeled data are re‐evaluated for their ability to further improve the classifier. Thus, active learning offers a solution to the problem of limited data annotations in pathology by having the pathologist engage actively with the algorithm, which evolves through continuous learning.

### Quality control and reliability of the algorithm

It is currently difficult to establish strict quality control steps for deep learning algorithms, especially in segmentation problems, for various reasons. A general principle in training any machine learning algorithm is to split the annotated data into ‘training’ and ‘test’ datasets, and ensure that these sets are independent when assessing performance. The algorithm should be trained on the training set and applied to the test set, then the results compared with the ‘ground truth’ associated to the test set. However, quality control for the segmentation step may suffer from the ‘**gold‐standard** paradox’ 8. This paradox arises from histopathological assessments by the pathologist being considered the gold standard, but the algorithm data may in fact be more reproducible than human assessment. This may be partially overcome by comparing the algorithm data to patient outcome, to see whether it is better able to predict outcome compared with manual pathology assessment/scoring. Still, the best methods to determine the reliability of an algorithm applied to novel datasets are an area of active debate.

In addition, local regulations apply to legally market any clinical‐grade software solution. In the USA, such an algorithm should be developed under the Food and Drug Administration (FDA)'s existing Quality System Regulation (QSR, 21 CFR Part 820), and Good Machine Learning Practices (GMLP; https://www.fda.gov/media/122535/download), which are currently being discussed.

### Understanding algorithms

A principal concern with the use of deep learning is that it is very difficult to understand some of the features and neural pathways used to make decisions. In particular, when deep learning is used to automatically extract features from an image that are directly correlated to clinical endpoints, without including a segmentation step where image objects are first extracted (see the section on correlating images to patient response), it is particularly challenging to understand why the algorithm reached its conclusions. Artificial neural networks have accordingly been described as a ‘**black box**’. This has led to several concerns: difficulty in correcting an underperforming algorithm; lack of transparency, explainability, and provability for humans who may not trust how an algorithm generates reliable results; and regulatory concerns because, unlike traditional image analysis, in deep learning the image features are abstracted in a way that is very difficult for a human to understand. In response, there have been efforts to convert deep learning algorithms into a ‘**glass box**’ by clarifying the inputs and their relation to measured outputs, making it more interpretable by a human using a variety of techniques 49, 50, 51, 53. By providing information to the reviewing pathologist about the histopathologic features used by the algorithm in a particular instance, trust in the algorithm can be fostered and a synergy between pathologist and machine can be achieved that may exceed the performance of either AI or pathologist alone 29.

### Ethics

The demand for personal health data has grown in the big data era. Researchers increasingly need to conduct studies using large amounts of data from disparate sources. This enables researchers to make novel connections that never could have been made before. Whether to publicly release data that went into creating an algorithm is a complex ethical problem. Providing transparency of the data used in developing an algorithm fosters interpretability, openness of scientific discovery, and increases acceptance and trust of the results. However, not exposing the data used by a deep learning model allows companies to create proprietary models that may not be validated or challenged in the public space. On the other hand, exposing private data of a patient (e.g. digital image with associated identifiable unique mutations) can present ethical concerns that violate privacy and as a result may prompt restrictive governance policies and security models. Another emerging issue relates to companies that leverage patient data in order to commercialize artificial intelligence tools and services. This raises ethical and legal concerns regarding data ownership and intellectual property rights. It is also important to have a system for data governance, which, among other aspects, controls who has access to what types and levels of data. Given the volume of data, their highly confidential nature, and the need to respect the rights of individuals – both for ethical reasons and to comply with the law – organizations should develop formal mechanisms to comprehensively address these issues, rather than address them *ad hoc*.

### Cyber‐security

Cyber‐security concerns of CPATH primarily stem from storing large amounts of medical data in cloud‐based systems that can be accessed via the internet. In order to minimize a data breach, it is prudent to decouple CPATH data (i.e. digital images) from patient data (i.e. personal identifiers such as medical record number and date of birth). Several cloud service providers now offer Health Insurance Portability and Accountability Act (HIPAA) compliant solutions. In addition, the FDA has created guidelines for cyber‐security (U.S. Food and Drug Administration, Postmarket Management of Cyber‐security in Medical Devices, 2016). The European Union's general data protection regulation (GDPR) 54 imposes similar security requirements on those who process personal data.

## Conclusion and future recommendations

Within the field of CPATH, the techniques for ML‐enhanced image analysis and its combination with other data sources have evolved to the point where they may soon be ready to be translated from the research environment into practicing clinical laboratories. Although the promises of CPATH are great, there are also manifold hurdles that need to be overcome 55. Regulators, vendors, and healthcare providers can all help to drive the field forward to benefit patients.

There are many ways in which regulation could be clarified around CPATH. Most importantly, the laboratory accreditation landscape must become flexible enough to accommodate novel scoring and deep learning methods that promise to improve accuracy and reproducibility 55, 57. Encouragingly, in April 2019, the FDA released a discussion paper on Good Machine Learning Practices (https://www.fda.gov/media/122535/download), proposing a new regulatory framework for continuously learning and ‘adaptive’ AI/ML algorithms by incorporating updates from real‐world use and experience. From a cyber‐security perspective, though current regulatory frameworks impose penalties for failure to protect data and set minimum standards, there should be clearer guidance on best practices.

Regulators and vendors should work together to set standards and increase interoperability of CPATH infrastructure components and software, and such standards should be formalized via regulatory guidance. Improved regulation would clarify the level of testing that vendors have to execute to show that the device is safe and effective, and benefit users who could combine different devices in their existing healthcare infrastructure. File format, compression, resolution reduction, selection of region size, and level of magnification could all impact compatibility and interoperability. A first step could be further developing a pathology‐specific digital imaging and communications in medicine (DICOM) standard 58, with associated tags that can be used in combination with an image. A next step could be normalizing images to allow interoperability; one of the standards that could be used is that of the International Color Consortium (ICC) profiles for visualization. Such standards should be used and encouraged across the digital pathology industry, on platform‐agnostic software that could run on any combination of operating systems and with any infrastructure architecture (further recommendations may be found in ref 7). Another step toward promoting the adoption of CPATH in routine pathology practice is to integrate AI tools with existing laboratory devices (e.g. whole slide scanners, auto‐stainers) and software systems such as the digital pathology platform, laboratory information system, and electronic medical records. Again, standardization will support this integration, for example working with the Integrating the Healthcare Enterprise (IHE) 59. Interoperability should improve integration between platforms to facilitate interaction and collaboration between pathologists across multiple laboratories as well as exchanging data with the goal to improve continuously learning algorithms. Such integration is crucial, since it is the pathologists who play the crucial role in human–computer interaction studies, finding the most efficient use of algorithms to maximize benefit for the diagnostic process, and identifying issues 60.

Finally, healthcare institutions could be encouraged to pool anonymized patient data and make them publicly accessible, so that researchers around the world may cooperate to develop more accurate diagnostic algorithms. Deep learning is a numbers game, and obtaining sufficient training data is often the primary hurdle, which can be addressed by collaborative data‐sharing initiatives, similar to what exists in biomedical imaging 61 but where digital pathology lags behind.

CPATH applications have the potential to transform the lives of patients, but it may still take a frustratingly long time. In order to capitalize sooner on the many benefits of adopting AI in pathology, we need to garner better cooperation among invested regulators, vendors, and healthcare providers.

## Author contributions statement

All the authors contributed to concepts in the paper, wrote portions of the text, revised the paper critically, and gave final approval for the version to be published.
